# Absolute estimation of initial concentrations of amplicon in a real-time RT-PCR process

**DOI:** 10.1186/1471-2105-8-409

**Published:** 2007-10-23

**Authors:** Marjo V Smith, Chris R Miller, Michael Kohn, Nigel J Walker, Chris J Portier

**Affiliations:** 1 Constella Group, Suite 200, 2605 Meridian Pky, Durham, NC, USA; 2 National Institute of Environmental Health Sciences, Research Triangle Park, NC, USA; 3 Now at Applied Biosystems Inc, USA; 4 Now deceased

## Abstract

**Background:**

Since real time PCR was first developed, several approaches to estimating the initial quantity of template in an RT-PCR reaction have been tried. While initially only the early thermal cycles corresponding to exponential duplication were used, lately there has been an effort to use all of the cycles in a PCR. The efforts have included both fitting empirical sigmoid curves and more elaborate mechanistic models that explore the chemical reactions taking place during each cycle. The more elaborate mechanistic models require many more parameters than can be fit from a single amplification, while the empirical models provide little insight and are difficult to tailor to specific reactants.

**Results:**

We directly estimate the initial amount of amplicon using a simplified mechanistic model based on chemical reactions in the annealing step of the PCR. The basic model includes the duplication of DNA with the digestion of Taqman probe and the re-annealing between previously synthesized DNA strands of opposite orientation. By modelling the amount of Taqman probe digested and matching that with the observed fluorescence, the conversion factor between the number of fluorescing dye molecules and observed fluorescent emission can be estimated, along with the absolute initial amount of amplicon and the rate parameter for re-annealing. The model is applied to several PCR reactions with known amounts of amplicon and is shown to work reasonably well. An expanded version of the model allows duplication of amplicon without release of fluorescent dye, by adding 1 more parameter to the model. The additional process is helpful in most cases where the initial primer concentration exceeds the initial probe concentration. Software for applying the algorithm to data may be downloaded at

**Conclusion:**

We present proof of the principle that a mechanistically based model can be fit to observations from a single PCR amplification. Initial amounts of amplicon are well estimated without using a standard solution. Using the ratio of the predicted initial amounts of amplicon from 2 PCRs is shown to work well even when the absolute amounts of amplicon are underestimated in the individual PCRs.

## Background

The powerful technique of cDNA amplification by polymerase chain reactions (PCR) is used to quantify small amounts of mRNA in a variety of applications from genomics to medicine[1,2]. Using reverse transcription, the mRNA is converted to cDNA, which is then amplified by repeated duplication in the PCR process (RT-PCR). Various detection systems are available, including Taqman probes and SYBR Green, which mark the amplification process by fluorescent emission and so allow monitoring the DNA amplification in real time. The Taqman system uses a sequence-specific probe that attaches to a nucleotide sequence within the template. After the attachment of primer and Taq enzyme, the duplication of the template strands proceeds in the extension step. As part of the extension, the attached part of the probe molecule is digested, separating the fluorescent reporter dye from the quencher sequence. Probe molecules are 'used up' in the duplication process. By contrast, the SYBR Green system binds non-specifically to any double-stranded DNA complex and emits fluorescence only in the attached state. The dye molecules dissociate during the denaturation step, but are used again in later cycles.

It is the goal of quantitative PCR to use the record of observed fluorescent emissions to estimate the initial amount of cDNA, either in absolute terms of the amount of cDNA at the start of the amplification or in relative terms when the initial amount of cDNA for one PCR is estimated as a fold change from that of another PCR. In either case, the estimation requires a mathematical expression or model to link the initial amount of amplicon to the observed fluorescence. The most well known approach uses data from the short span of cycles whose fluorescent emission just rise above the background fluorescence, but for which duplication can still be approximated by an exponential curve, such as the one below:

*F*_*C*_= *F*_0_(1 + *E*)^*C*^

Here *F*_*C *_is the observed fluorescence at the C^th ^thermal cycle, and F_0_, the fluorescence that would be emitted by the initial amount of amplicon, when duplicated. The quantity *F*_*C *_is generally found to be less than *F*_0 _*2*^*C*^, so that, using equation 1, the efficiency, *E*, can be no greater than 1.

Many different and ingenious approaches have been published using this expression; however, the use of this expression brings with it 2 important drawbacks:

1) The observations of relatively few cycles are used, and those cycles are not always easy to identify.

2) Information beyond the observations from the PCR amplifications is needed to convert from the observed fluorescent scale to estimated quantities of amplicon in nmol/L.

The 2^nd ^drawback is of importance only for the estimation of absolute quantities of amplicon and does not apply to methods of relative quantitation [3,4].

Absolute quantitation using this principal is achieved using the standard curve technique [5], where the efficiency of the duplication is found by amplifying serial dilutions of a known concentration of the amplicon. This approach can be costly both in time taken to construct the standard solutions and in reagents for the PCR reactions since multiple samples are used. Alternatively, Swillens et al applied equation 1 specifically to PCRs run with the Taqman probe, making use of the fact that the probe is used up in amplification process [6]. By assuming that the entire initial amount of probe is used up and relating the initial probe amount to the final fluorescent reading, they estimated the conversion factor between number of probe molecules and observed fluorescence, thus estimating the initial amount of amplicon absolutely.

The effort to use more of the thermal cycles in a PCR has led to fitting a mathematical expression for a sigmoid curve to all of the fluorescent data using nonlinear regression [7-9]. The curves used by these authors can fit the observations very well, but do not reflect any of the internal processes of a PCR, so that it is difficult to fit them more precisely by adding information that may be known about specific reactants. There is also no intrinsic reason to believe that a curve that fits observations above the background fluorescence will also fit the observations below the background fluorescence, which could also affect the estimates of the initial amount of amplicon. Furthermore, when the sigmoid curves are applied to a dye that is not inactivated by the duplication process, a standard solution with a known amount of the target molecules is still needed to estimate the conversion factor between fluorescent emission and number of target molecules [7,8]. Goll et al applied numerous sigmoid expressions to observations generated by the Taqman probe but again used solutions with known copy number to estimate the conversion factor [9].

Alternatively, detailed models of the reactions taking place in the anneal and extension steps of the PCR have also been presented [10,11]. Using reaction equations to describe each chemical reaction in the duplication process, a detailed picture is built up of what is known of the process. Including all reactions allows insight into consequences of limiting nucleotide bases, primers or enzyme for example, but requires a large number of parameters that are approximated with values from the literature. The models describe the chemical processes in a PCR consistent with detection systems similar to SYBR Green, so that there are no equations comparable to the annealing and digestion of the Taqman probe. This simplifies the system of equations, but again requires the information of a standard solution to estimate the conversion factor between fluorescent emission and number of target molecules.

In this paper, we present a compromise between the last two approaches. That is, we present a model based on the chemical reactions taking place in the anneal step of PCRs with Taqman probe, but use simplifying assumptions, to reduce the model to only 3 parameters. All parameters are shown to be estimable using the observations from a single amplification. Since the model includes the digestion of probe molecules as part of the duplication, a more precise estimate of the amount of probe converted to fluorescence and therefore a more precise estimate of the conversion factor is possible.

In the body of the paper we present the basic model in detail, including the duplication process with probe digestion as well as the re-annealing of template. In the Appendices we present equations that represent an additional process that may be important to particular PCRs: duplication of template without release of fluorescence, *i.e*. 'unmarked duplication'.

We fit the model to 3 data sets with known initial concentrations of amplicons to evaluate the model's estimates. Adding the unmarked duplication process improved fits and estimates in cases where the initial amount of probe was smaller than the initial amount of primer. For one of the data sets the estimates are quite good, and in all cases the estimates are of the right order of magnitude, though sometimes underestimated. In the cases where the initial amount of amplicon is underestimated, the fold changes of different concentrations of the amplicon are still well estimated, indicating that a factor common to both amplifications has been left out of the model.

## Results

### General description of model

The model presented in this paper estimates the initial amount of template that is amplified through the PCR process. If the PCR process is used to quantify an unknown amount of mRNA (as opposed to an unknown amount of cDNA directly), the mRNA must first be reverse transcribed to form what is in theory an equivalent amount of cDNA. The efficiency of the transcription varies with the practitioner and the reagents, but estimating the efficiency of the transcription is beyond the scope of this paper. It is the transcribed amount of cDNA that is estimated by our approach.

Once the mRNA has been transcribed, the PCR process acts to duplicate the cDNA through a succession of thermal cycles. The thermal cycles alternate between 2 phases: the anneal/extension step and the denaturation step. Ideally in the anneal/extension step, each single strand of template is hybridized by both a probe molecule and a primer molecule. When a molecule of Taq enzyme attaches to such a triplex, the extension process begins and nucleotide bases attach to form a duplex consisting of the original DNA template and a completed second strand of opposite orientation. As part of the extension process, the Taq enzyme also digests the attached part of the Taqman probe, releasing and separating the fluorescent marker from the quencher sequence. A given Taqman probe only attaches to DNA sequences of a particular orientation. For the data in this paper, the probe molecules were designed to attach to molecules of the DNA sequences corresponding to the cDNA transcribed from the original sample of mRNA. The single-stranded DNA of opposite orientation duplicates without probe attachment and so without fluorescent emission. We assume duplication of strands of either orientation proceeds at the same rate. We also assume all duplexes separate during the hotter denaturation step.

The simplified model presented in this paper, includes an approximation to the above duplication process, predicting the amounts of both probe and primers used by the PCR for each cycle. In addition, the model includes the process of re-annealing, whereby single strands of DNA previously synthesized and of opposite orientation hybridize. The competition for single strands of DNA by this second process diminishes the predicted efficiency of the duplication process over the course of the PCR [10,12]. Details are shown in the Methods section. We show the results below of the basic model and the model extended by adding the unmarked duplication process.

**Figure 1:**
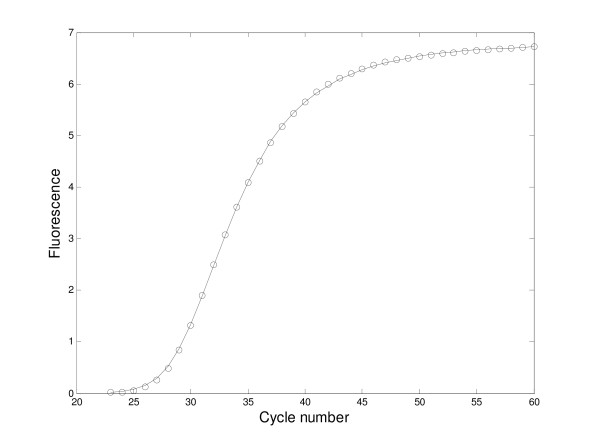
**Model fit**. The observations and predicted values are shown corresponding to one of the amplifications using RnaseP. For this fit, the true initial amount of amplicon was 1.667e-7 nmol/L (in 50 μl solution) and the estimated parameter values as follows: the estimated concentration of amplicon was 1.767e-7 nmol/L, the estimated re-annealing rate constant, 1.1680e4; the estimated rate constant for unmarked duplication, 266.21; and the estimated conversion factor, 2.9392e-2. The predicted values correspond to the equilibrium values of the solution to the 1^st ^equation in the extended model, the *w*_*iqE*_. See Appendix.)

The model was applied to 3 data sets described in the method section. The 3 data sets amplified a total of 3 gene sequences, RnaseP, Cyp1B1 and Actin. In each case, the initial concentrations of the amplicon were known, with amplifications run on several known dilutions of the original concentration. Tables 1 through 4 in the body of the paper compare the estimated amount of amplicon with the known concentration. Figure 1 shows an example of the model fit to an amplification run of the RNaseP data set.

**Table 1:** RNaseP estimates including replicates

	predictions from basic model		predictions from expanded model	
Initial number of amplicon molecules	40 cycles	60 cycles	40 cycles	60 cycles

1.25 × 10^3^	0.712 × 10^3^	0.866 × 10^3^	0.821 × 10^3^	1.20 × 10^3^
	0.734 × 10^3^	0.900 × 10^3^	0.831 × 10^3^	1.25 × 10^3^
	0.723 × 10^3^	0.888 × 10^3^	0.833 × 10^3^	1.20 × 10^3^
	0.734 × 10^3^	0.887 × 10^3^	0.830 × 10^3^	1.23 × 10^3^
2.5 × 10^3^	1.50 × 10^3^	1.8 × 10^3^	1.7 × 10^3^	2.4 × 10^3^
	1.5 × 10^3^	1.8 × 10^3^	1.7 × 10^3^	2.5e × 10^3^
	1.6 × 10^3^	2.0 × 10^3^	1.9 × 10^3^	2.7 × 10^3^
	1.7 × 10^3^	2.1 × 10^3^	2.0 × 10^3^	2.9 × 10^3^
5.0 × 10^3^	3.3 × 10^3^	3.9 × 10^3^	3.7 × 10^3^	5.1e × 10^3^
	3.2 × 10^3^	3.7 × 10^3^	3.6 × 10^3^	5.1e × 10^3^
	3.2 × 10^3^	3.7 × 10^3^	3.6 × 10^3^	5.3 × 10^3^
	3.0 × 10^3^	3.3 × 10^3^	3.5 × 10^3^	5.0 × 10^3^
1.0 × 10^4^	0.61 × 10^4^	0.66 × 10^4^	0.71 × 10^4^	1.2 × 10^4^
	0.60 × 10^4^	0.67 × 10^4^	0.83 × 10^4^	1.2e × 10^4^
	0.61 × 10^4^	0.67 × 10^4^	0.72 × 10^4^	1.2e × 10^4^
	0.61 × 10^4^	0.66 × 10^4^	0.71 × 10^4^	1.1 × 10^4^
2.0 × 10^4^	1.3 × 10^4^	1.3e × 10^4^	2.0 × 10^4^	2.4 × 10^4^
	1.3 × 10^4^	1.4 × 10^4^	1.5 × 10^4^	2.3 × 10^4^
	1.3 × 10^4^	1.5 × 10^4^	1.8 × 10^4^	2.4e × 10^4^
	1.4 × 10^4^	1.6 × 10^4^	2.2 × 10^4^	2.5 × 10^4^

Estimation results of initial amounts of RNaseP amplicon. Initial concentrations of probe and primer were 250 nmol/L and 900 nmol/L respectively. The basic model accounts for the duplication of target sequence with release of fluorescence as well as re-annealing of previously formed DNA strands. The extended model additionally takes into account duplication of target sequence without probe attachment and hence without release of fluorescence. Results are given for individual replicates of each initial amount of amplicon.

**Table 2:** Cyp1B1 estimates with replicates

	predictions from basic model		predictions from expanded model	
Initial number of amplicon molecules	40 cycles	60 cycles	40 cycles	60 cycles

1e6	0.59 × 10^6^	0.60 × 10^6^	0.66 × 10^6^	0.67 × 10^6^
	0.56 × 10^6^	0.58 × 10^6^	0.63 × 10^6^	0.63 × 10^6^
	0.56 × 10^6^	0.59 × 10^6^	0.65 × 10^6^	0.64 × 10^6^
1e5	0.48 × 10^5^	0.50 × 10^5^	0.55 × 10^5^	0.54 × 10^5^
	0.48 × 10^5^	0.52 × 10^5^	0.56 × 10^5^	0.58 × 10^5^
	0.53 × 10^5^	0.55 × 10^5^	0.61 × 10^5^	0.61 × 10^5^
1e4	0.46 × 10^4^	0.50 × 10^4^	0.47 × 10^4^	0.54 × 10^4^
	0.45 × 10^4^	0.49 × 10^4^	0.47 × 10^4^	0.53 × 10^4^
	0.50 × 10^4^	0.55 × 10^4^	0.52 × 10^4^	0.59 × 10^4^
1e3	0.52 × 10^3^	0.59 × 10^3^	0.57 × 10^3^	0.63 × 10^3^
	0.48 × 10^3^	0.53 × 10^3^	0.52 × 10^3^	0.58 × 10^3^
	0.51 × 10^3^	0.57 × 10^3^	0.57 × 10^3^	0.61 × 10^3^

Estimation results of initial amounts of Cyp1B1 amplicon. Initial concentrations of probe and primer were 100 nmol/L and 200 nmol/L respectively. The basic model accounts for the duplication of target sequence with release of fluorescence as well as re-annealing of previously formed DNA strands. The extended model also takes into account duplication of target sequence without probe attachment and hence without release of fluorescence. Results are given for individual replicates of each initial amount of amplicon.

**Table 3:** Comparison of fold change estimates for Cyp1B1 and RNaseP

Cyp1B1 means of 3 estimates with std error					
	basic model		expanded model		
	
true fold change	40 cyc	60 cyc	40 cyc	60 cyc	Livak et al

1e6/1e5 = 10	11.5 (0.504)	11.3 (0.374)	11.3 (0.389)	11.3 (0.587)	14.0 (1.08)
1e6/1e4 = 100	121.6 (4.91)	115.2 (4.00)	133.2 (4.48)	117.1 (4.59)	112.2 (4.81)
1e6/1e3 = 1000	1133 (19.83)	1049 (23.4)	1170 (21.4)	1066 (10.78)	1267 (30.9)

RNaseP means of 4 estimates with std error					

	basic model		expanded model		
	
true fold change	40 cyc	60 cyc	40 cyc	60 cyc	Livak et al

2e4/1e4 = 2	2.2 (.039)	2.2 (.098)	2.6 (0.278)	2.0 (0.078)	2.0 (.043)
2e4/5e3 = 4	4.2 (.0164)	4.0 (.032)	5.2 (0.44)	4.7 (0.114)	4.1 (.083)
2e4/2.5e3 = 8	8.4 (.142)	7.5 (0.117)	10.3 (0.677)	9.2 (0.299)	9.0 (.0678)
2e4/1.25e3 = 16	18.3 (0.295)	16.4 (0.681)	22. (1.83)	19.7 (0.434)	19.1 (.419)

The estimates of the initial amounts of amplicon from Tables 1 and 2 were used to estimate the fold change from the solutions with the largest initial amounts of amplicon to those with lower amounts. The first column gives the correct fold change. The final column shows fold change estimates using the method described by Livak et al [3](See text.).

**Table 4:** Estimates from averaged data

	Cyp1B1.			Actin	
	
	AmpliconCount	Basic Model	Expanded Model	Basic Model^2^	Expanded Model^3^
ABI Master Mix primer = 400 nmol/L probe = 400 nmol/L	10^3^	0.78 × 10^3^	0.68 × 10^3^	0.25 × 10^3^	0.24 × 10^3^
	10^4^	0.71 × 10^4^	0.64 × 10^4^	0.36 × 10^3^	0.35 × 10^3^
	10^5^	0.86 × 10^5^	0.77 × 10^5^	0.48 × 10^4^	0.47 × 10^4^
	10^6^	1.0 × 10^6^	0.94 × 10^6^	0.59 × 10^5^	0.57 × 10^5^

ABI Master Mix primer = 400 nmol/L probe = 200 nmol/L	10^3^	0.48 × 10^3^	0.52 × 10^3^	0.26 × 10^3^	0.28 × 10^3^
	10^4^	0.48 × 10^4^	0.52 × 10^4^	0.39 × 10^4^	0.41 × 10^4^
	10^5^	0.62 × 10^5^	0.64 × 10^5^	0.37 × 10^5^	0.40 × 10^5^
	10^6^	0.77 × 10^6^	0.78 × 10^6^	0.45 × 10^6^	0.47 × 10^6^

ABI Master Mix primer = 200 nmol/L probe = 400 nmol/L	10^3^	0.50 × 10^3^	0.48 × 10^3^	0.27 × 10^3^	0.23 × 10^3^
	10^4^	0.54 × 10^4^	0.46 × 10^4^	0.46 × 10^4^	0.38 × 10^4^
	10^5^	0.76 × 10^5^	0.70 × 10^5^	0.53 × 10^5^	0.48 × 10^5^
	10^6^	0.90 × 10^6^	0.86 × 10^6^	0.67 × 10^6^	0.58 × 10^6^

ABI Master Mix primer = 200 nmol/L probe = 200 nmol/L	10^3^	0.33 × 10^3^	0.32 × 10^3^	0.20 × 10^3^	0.20 × 10^3^
	10^4^	0.34 × 10^4^	0.33 × 10^4^	0.26 × 10^4^	0.26 × 10^4^
	10^5^	0.42 × 10^5^	0.42 × 10^5^	0.30 × 10^5^	0.29 × 10^5^
	10^6^	0.53 × 10^6^	0.53 × 10^6^	0.39 × 10^6^	0.38 × 10^6^

Estimation results of initial amounts of Cyp1B1 and Actin amplicons. Initial concentrations of probe and primer are given in the first column. Note that using the extended model only increases the estimates for the PCRs with a higher initial primer concentration than probe. ^1^Initial number of amplicon molecules placed in solution. ^2^Equations found in Table 2. ^3^Equation (S1) in the Appendix.

### Model choice and cycle number

In Table 1, we present the estimation results of the initial amounts of amplicon for the RNaseP data set. As PCRs are frequently run for 40 rather than 60 cycles, the results are analyzed using just the first 40 cycles as well as using all available 60 cycles. Both the basic model and the extended model (allowing for duplication of the target sequence without release of fluorescence; see Appendix (additional file 1)) were used.

**Figure 2:**
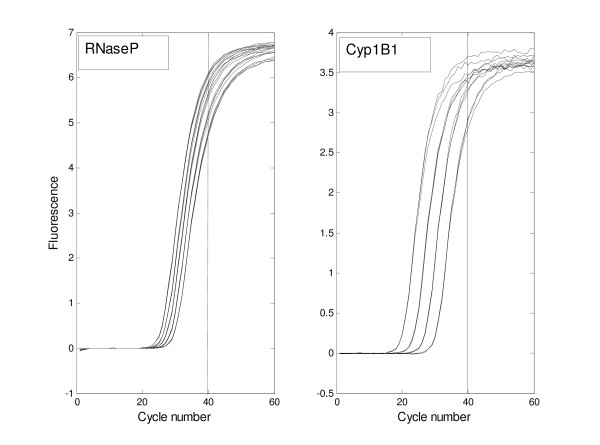
**PCR results for RNaseP and Cyp1B1, showing replicates**. Observations from the RNaseP and Cyp1B1 data sets are shown in linear scale, with the 40 cycle cutoff marked by the vertical line. The horizontal axes show cycle number.

Even when the basic model is used with the data of only 40 cycles (2^nd ^column), the estimates of the initial concentration of amplicon are of the right order of magnitude (although substantially underestimated), ranging from 57% to 72% of the true value. The estimates are somewhat better for the larger initial amounts, (63 – 72% of the true value) than for the smallest initial amounts (57 – 59% of the true value), suggesting that there is information carried in the later cycles of a PCR. Figure 2A shows that the measured fluorescence at the 40^th ^cycle is further from the horizontal asymptote for the lowest initial amounts of amplicon so that there are fewer information-carrying observations for the lower dilutions.

The results of using the basic model with all 60 cycles are shown in the 3^rd ^column of Table 1 and range from 66% of the true value to 85%. For these data the estimates do not seem to improve with higher initial concentrations, suggesting that 60 cycles were sufficient for these initial amounts of amplicon. When the extended model is used, even the estimates using only 40 thermal cycles improve consistently, with the quality of the estimate again increasing with the initial concentration. Using the expanded model with 60 cycles leads to some overestimation for the highest initial concentration, but generally estimates initial amounts of amplicon very close to the true values.

In Table 2, the same patterns seen in Table 1 are repeated: the basic model with 40 cycles shows the poorest estimates, especially for the lowest initial concentrations. Adding the last 20 cycles improves all estimates, but the improvement is greater for the lower initial concentrations. The estimates are again of more uniform quality for the basic model applied to the data from all 60 cycles. Allowing for duplication of target sequence without fluorescent emission (i.e., using the expanded model) again improves the estimates using both 40 and 60 thermal cycles of data. For this data set all estimates underestimated the true initial amount of amplicon, estimating values between 53% and 67% of the true value.

### Fold change prediction

In Table 3, we use the absolute estimates found earlier to estimate fold changes between dilutions of the initial amounts of amplicon. In each case we compared the highest concentration to a lower one. For the Cyp1B1 data, this meant computing 3 estimates of a fold change, while we were able to compute 4 estimates of each fold change for the RNaseP data set.

In contrast to the absolute estimates, the estimates of the fold changes are not improved by using the expanded model over the basic model. The best estimates generally come from using 60 cycles of data with the basic model, which provides the largest amount of data with the fewest parameters to be estimated. The conclusion is that the basic model may underestimate the amount of amplicon by a consistent factor over the various concentrations, so that the fold change remains well estimated. In that case having fewer parameters to estimate with more data provides better estimates. Correspondingly, the worst estimates of the fold change are made using the expanded model with 40 cycles of data.

Fold change estimates made using the 2−ΔCT
 MathType@MTEF@5@5@+=feaafiart1ev1aaatCvAUfKttLearuWrP9MDH5MBPbIqV92AaeXatLxBI9gBaebbnrfifHhDYfgasaacH8akY=wiFfYdH8Gipec8Eeeu0xXdbba9frFj0=OqFfea0dXdd9vqai=hGuQ8kuc9pgc9s8qqaq=dirpe0xb9q8qiLsFr0=vr0=vr0dc8meaabaqaciaacaGaaeqabaqabeGadaaakeaacqaIYaGmdaahaaWcbeqaaiabgkHiTiabfs5aejabdoeadnaaBaaameaacqWGubavaeqaaaaaaaa@328B@ method [3] are listed in the final column of Table 3 for comparison. In this approach a 'threshold value' is set for fluorescent emission, low enough that amplification is still exponential. The dilution fold is estimated using the fractional cycle numbers (CT) at which the amplifications cross that threshold in the following formula:

Fc=2−[CThigh−CTlow]
 MathType@MTEF@5@5@+=feaafiart1ev1aaatCvAUfKttLearuWrP9MDH5MBPbIqV92AaeXatLxBI9gBaebbnrfifHhDYfgasaacH8akY=wiFfYdH8Gipec8Eeeu0xXdbba9frFj0=OqFfea0dXdd9vqai=hGuQ8kuc9pgc9s8qqaq=dirpe0xb9q8qiLsFr0=vr0=vr0dc8meaabaqaciaacaGaaeqabaqabeGadaaakeaacqWGgbGrcqWGJbWycqGH9aqpcqaIYaGmdaahaaWcbeqaaiabgkHiTmaadmaabaGaem4qamKaemivaq1aaSbaaWqaaiabdIgaOjabdMgaPjabdEgaNjabdIgaObqabaWccqGHsislcqWGdbWqcqWGubavdaWgaaadbaGaemiBaWMaem4Ba8Maem4DaChabeaaaSGaay5waiaaw2faaaaaaaa@4394@

Here *Fc *is the estimated fold change. The subscripts 'high' and 'low' refer respectively to the highest initial concentration of amplicon and the lower concentration being compared to it. The threshold was set at 10 times the standard deviation of the first 10 cycles of the highest concentration in the comparison. Generally, the estimates made using all 60 cycles with the basic model are closer to the true fold changes.

### Varying probe and primer concentrations

The last data set comprises the averaged observations over 3 replicates of several PCRs applied to the Cyp1B1 and Actin sequences with various combinations of initial probe and primer concentrations. The results are shown in Table 4. The 1^st ^column gives the initial concentrations of probe and primers; the 2^nd ^column gives the initial number of amplicon molecules. Columns 3 and 4 give the results for the Actin sequence; the last 2 columns give the results for the Cyp1B1 sequence.

The results vary between probe/primer combinations and gene sequence, with good results for Cyp1B1 generally for the higher concentrations. The initial amounts of Actin amplicon are considerably underestimated, although these estimates also improve for higher initial amounts. Nonetheless there is a consistent pattern that shows some improvement of the estimates using the extended model, but only for those cases where the initial concentration of primer exceeded the initial concentration of probe. This is consistent with the improvement in estimates seen with the extended model in Tables 1 and 2, both of which also show results for amplifications with more primer than probe available. For every estimate in Table 4 where the concentration of probe is at least as high as that of primers, the basic model either outperforms the expanded model or fails to be improved by the extended model. We conclude that as long as there is at least as much probe as primer available, the preferential attachment of template to probe over primer precludes duplication of template without probe attachment. In that case, the model with the fewer parameters will provide the better estimate, since the extra parameter may be over-fitting to noise.

## Discussion

In this paper we explore balancing mechanistic modelling of the chemical reactions taking place in a PCR with the requirement that the model be estimable using the data from a single PCR amplification without an additional standard solution. The model was developed using Taqman probes to monitor the duplication. The Taqman detection system worked well, because the probe molecules are digested in the course of duplication. This characteristic allows the estimation of the conversion factor between the number of digested probe molecules and the observed fluorescence. While others have used this characteristic before [6], they required that all the probe molecules be used up. By modelling the consumption of probe molecules, the conversion factor can in principle by calculated using the predicted number of digested probe molecules and comparing that to the observed increase in fluorescence.

The presence of unused probe molecules after the completion of the PCR was predicted by the extended model in both data sets containing replicate data (RnaseP and Cyp1B1). In both of these data sets more primer was available than probe, but with both re-annealing and unmarked duplication included in the model, unused probe was left even though the upper asymptote was clearly reached (Fig 2). Between 82% and 93.5% of probe was used in the RnaseP amplifications and around 95% for the Cyp1B1 amplifications.

In applying the model to the data, we found that the observed upper asymptotes corresponding to identical combinations of initial amounts of probe and primers did not always have the same values. This is visible in Fig. 2B, showing the Cyp1B1 replicate data. We were unable to reproduce the differences in the upper asymptotes without allowing different conversion factors between observed fluorescence and the number of digested probe molecules. For this reason we did not use a common conversion factor.

The results of the estimation of amplicon were mixed. For the RNaseP amplifications, the estimation of the initial amount of amplicon was quite good; for the Cyp1B1 and the Actin sequences the initial amount of amplicon was underestimated, sometimes severely so. Even in these cases, the fact that the estimates were of the right order of magnitude indicate that the approximations used in the model were reasonable. The underestimations do indicate that more work needs to be done with this approach before it can be used by itself or in a clinical setting.

In order to simplify the chemical reactions taking place in the PCR to the point that the model could be estimated by a single amplification means that many processes are necessarily left out. The cumulative effect of all unmodeled processes could be significant, even if the individual unmodeled reactions have a small effect on the outcome. We mention here several of the reactions that were not included in the results presented here.

Primer dimerization occurs when primers specific to DNA strands of opposite orientation hybridize during the anneal step. Primer-dimers may extend during the extension step, in which case the primers are no longer usable, even after denaturation. If the dimers do not extend, then the primers may be used in later cycles [11]. Although a differential equation describing primer-dimerization can be constructed (See Appendix A (additional file 1)), the problem is that the additional parameter has to be estimated. If primer-dimerization is added to the extended model, then a total of 5 parameters have to be estimated for a single amplification, which is sometimes too many. We did attach the primer-dimerization equation to the basic model and applied the result to some of the amplifications of the averaged data. For these data, adding the additional reaction improved neither the fit nor the estimate.

The amount of Taq enzyme might become limiting, if the enzyme degrades over the course of the PCR as a result of repeated exposures to high temperatures. The decrease in enzymatic activity has been investigated [10] and did not seem to have a strong impact on the amplification process.

In simplifying the model, we were especially ruthless with the extension step, assuming perfect irreversible extension for all cases. This means we did not examine the quantity of nucleotides being limiting or the possibility of incomplete extension [11]. Unlike the primer-dimerization, these processes would be very difficult to incorporate into the model, and still have it be estimable from a single amplification.

In spite of these shortcomings however, the model (both the basic and the extended models) did prove to be estimable in all cases. The predictions when compared to the known concentrations of amplicon were quite good in the case of the extended model applied to the RnaseP sequence and of the right order of magnitude (though underestimated) in the other cases. Furthermore, the fold change estimates using the basic model did well for both the RnaseP and the Cyp1B1 sequence. In addition, the model seems to be sensitive enough that processes included in the model that do not contribute to the output of a particular amplification also do not improve the fit. In particular, this was the case for the unmarked duplication process where enough probe was available (Table 4).

## Conclusion

There are advantages to having a model that can estimate initial quantities of amplicon from a single amplification; using only the data from a single amplification means that no additional reference tissues need to be analyzed, no standard solutions need to be made up, no additional replicates or dilutions need to be run, and there are no concerns about whether the efficiencies of the amplifications or the conversion factors linking fluorescent emissions to molecules are nearly enough the same across replicates or reference solutions. There are also advantages to using a model based on the science of the process: such a model allows the estimated quantities to have meaning within the context of the chemical reactions, provides a basis for comparison across experiments run under differing conditions and allows greater insight into the critical components that govern the net outcome of a complicated series of reactions.

In this paper we present proof of the principle that it is possible to estimate absolute concentrations of template in single PCR amplifications through modeling the biochemical processes present within the thermal cycles. That the drastically simplified model nonetheless produced good estimates in some cases and at least estimates of the right order of magnitude in others shows that the assumptions going into the model produce good approximations.

## Methods

### Data

PCR quantification was applied to solutions with known concentrations of human gene sequences from 3 data sets. The PCRs in each data set were run for 60 thermal cycles using 50 μl wells. Cycles consisted of anneal/extension steps (each lasting 60 seconds at 60°C) alternating with denature steps (each lasting 15 seconds at 95°C). The first data set consists of 20 PCRs run on the RnaseP sequence and includes 4 replicates of each of 5 initial amounts of amplicon (1250, 2500, 5 × 10^3^, 10^4^, and 2 × 10^4 ^molecules). The probe and primers were supplied by an AB Biosystems pre-packaged kit. For this data set a 7900 model machine was used with a 96 well plate and a kit with part number 4310982. (Identical probe and primers for models 7300 or 7500 come in part number 4350584.) The initial primer concentrations were all 900 nmol/L; the initial probe concentrations, all 250 nmol/L.

The second data set consists of 12 PCRs on Cyp1B1, including 3 replicates of each of 4 initial concentrations of amplicon (10^3^, 10^4^, 10^5^, and 10^6 ^molecules). The primers for both orientations of each sequence were found using the Primer Express software program (Applied Biosystems). The forward primer used was GCC AGC CAG GAC ACC CT with the reverse primer, GAT CCA ATT CTG CCT GCA CTC. The probe used was CGC TTGC AGT GGC TGC TCC TCC T. The Taqman probe carried a FM reporter label with Tamra as quencher. The initial primer concentrations were 200 nmol/L; the initial probe concentrations, 100 nmol/L.

The final data set contains PCRs run on both Cyp1B1 and Actin sequences with 4 different combinations of initial probe and primer concentrations. The primer sequences used were again found using the Primer Express software program (Applied Biosystems). For the Actin sequence, the forward primer was CCT GGC ACC CAG CAC AAT and the reverse primer was GCT GAT CCA CAT CTG CTG GAA. The probe used was ATC AAG ATC ATT GCT CCT CCT GAG CGC with the VIC reporter label and a Tamra quencher. The 4 combinations of initial probe and primer concentrations used for both gene sequences were 400 nmol/L probe with 400 nmol/L primer, 400 nmol/L probe with 200 nmol/L primer, 200 nmol/L primer with 400 nmol/L probe and 200 nmol/L for both probe and primer. For each combination of probe and primer 12 PCRs were run with 3 replicates of 4 initial concentrations of amplicon (3.32 × 10^-5 ^nmol/L, 3.32 × 10^-6 ^nmol/L,, 3.32 × 10^-7 ^nmol/L, and 3.32 × 10^-8 ^nmol/L, all in 50 μl of solution.) Only the mean observations, averaged over the 3 replicates were available.

### Model development

The model provides a simplified description of the duplication of DNA template using the Taqman probe fluorescent detection system. We focus on reactions taking place in the anneal step of the PCR amplification. The attachment of Taq enzyme and subsequent extension of complexes formed in the anneal step is assumed to follow immediately upon the attachment of primer and is therefore not separately modeled. Similarly, we assume that during the denaturation or melting step all duplexes 'melt' or separate into single strands, so that these reactions are also not separately modeled. The reactions that form the focus of the basic model are given in Table 5. The DNA template is referred to as *A *with the sequence specific primer, *P*. The anti-sense counterparts are denoted by primes. The Taqman probe molecule is assumed to hybridize only with *A *(not *A'*) and is denoted by *Q*.

**Table 5:** Reactions in the anneal step incorporated in the basic model

	[A] + [Q] ↔ [AQ]
	[AQ] + [P] ↔ [AQP]
	[A'] + [P'] ↔ [A'P']
	[A'] + [A] → [A'A]

The reactions listed in Table 5 are shown as reversible, with the exception of the re-annealing of existing strands of DNA leading directly to the duplex, *A'A*. As mentioned by other authors [10], the duplex *A'A *rarely dissociates. If the reactions as they appear in Table 5 are allowed to go to equilibrium, very little product will be formed, as the re-annealing reaction would eventually use up all the single DNA strands. In their paper, Gevertz et al [10] therefore consider all reactions as being stopped by the end of the anneal/extension step, rather than letting all reactions go to equilibrium. However, when PCR runs with anneal/extension steps varying from 30 seconds to 90 seconds were compared, no discernible differences were observed (not shown). From this we concluded that the reactions in an anneal/extension step do go to equilibrium very quickly.

Our solution is to assume that although the reactions attaching probe or primer are themselves reversible, the attachment of a primer molecule is followed very quickly by the attachment of Taq enzyme. We further assume that extension follows quickly and irreversibly and continues until the duplex is made. In effect then, the reactions in Table 5 forming the triplex, *AQP*, and the complex, *A'P'*, are irreversible and can compete with the re-annealing reactions, even if the reactions are all allowed to go to equilibrium.

Table 5 does not contain the reaction attaching primer *P *to template *A*. For the basic model, we assume that the probe molecules are designed to attach more quickly to the template than the primers, so that the rate-limiting step is that of the attachment of primer, immediately followed by enzyme attachment and extension. We relax this assumption in the extended model discussed in more detail in the Appendix A(additional file 1). If the rate-limiting step is the attachment of the primer rather than the probe, we can also assume that double-stranded DNA formed from the complex, *A'P'*, is produced at the approximately the same rate as that from the complex, *AQP*. For this reason, the annealing and extension processes associated with the anti-sense strands, *A'*, are also not modeled separately, but assumed to proceed at the same rate as for the sense strands, *A*.

Table 6 shows the differential equations actually making up the basic model. The subscript i refers to the anneal step in the i^th ^thermal cycle. Thus, *A*_*i*_, *Q*_*i *_and *P*_*i *_refer to the amounts of template, probe and primer available at the start of the i^th ^anneal step. All quantities in the equations in Table 6 are in units of nmol/L, since that is how the probe and primer are measured. The 1^st ^equation in Table 6 approximates the result of the first two reactions in Table 5. The intermediate product of the 1^st ^reaction, *AQ*, is assumed to be short-lived, progressing quickly to *AQP *with the attachment of a primer. The solution to the 1^st ^equation, *w*_*iq*_(*t*), then reflects the amount of double-stranded DNA formed by attachment of probe and primer to template with subsequent extension. The factors (*Q*_*i *_- *w*_*iq*_(*t*)) and (*P*_*i *_- *w*_*iq*_(*t*)) refer to the amounts of probe and primer respectively available at time t within the i^th ^anneal step, being the amount of probe and primer present at the start of the i^th ^anneal step minus the amounts already used in duplications by time t.

**Table 6:** The differential equations making up the basic model

dwiq(t)dt=Mq[Ai−wiq(t)−wir(t)]∗[Pi−wiq(t)]∗[Qi−wiq(t)] MathType@MTEF@5@5@+=feaafiart1ev1aaatCvAUfKttLearuWrP9MDH5MBPbIqV92AaeXatLxBI9gBaebbnrfifHhDYfgasaacH8akY=wiFfYdH8Gipec8Eeeu0xXdbba9frFj0=OqFfea0dXdd9vqai=hGuQ8kuc9pgc9s8qqaq=dirpe0xb9q8qiLsFr0=vr0=vr0dc8meaabaqaciaacaGaaeqabaqabeGadaaakeaadaWcaaqaaiabdsgaKjabdEha3naaBaaaleaacqWGPbqAcqWGXbqCaeqaaOWaaeWaaeaacqWG0baDaiaawIcacaGLPaaaaeaacqWGKbazcqWG0baDaaGaeyypa0Jaemyta00aaSbaaSqaaiabdghaXbqabaGcdaWadaqaaiabdgeabnaaBaaaleaacqWGPbqAaeqaaOGaeyOeI0Iaem4DaC3aaSbaaSqaaiabdMgaPjabdghaXbqabaGcdaqadaqaaiabdsha0bGaayjkaiaawMcaaiabgkHiTiabdEha3naaBaaaleaacqWGPbqAcqWGYbGCaeqaaOWaaeWaaeaacqWG0baDaiaawIcacaGLPaaaaiaawUfacaGLDbaacqGHxiIkdaWadaqaaiabdcfaqnaaBaaaleaacqWGPbqAaeqaaOGaeyOeI0Iaem4DaC3aaSbaaSqaaiabdMgaPjabdghaXbqabaGcdaqadaqaaiabdsha0bGaayjkaiaawMcaaaGaay5waiaaw2faaiabgEHiQmaadmaabaGaemyuae1aaSbaaSqaaiabdMgaPbqabaGccqGHsislcqWG3bWDdaWgaaWcbaGaemyAaKMaemyCaehabeaakmaabmaabaGaemiDaqhacaGLOaGaayzkaaaacaGLBbGaayzxaaaaaa@6D36@
dwir(t)dt=Mr[Ai−wiq(t)−wir(t)]2 MathType@MTEF@5@5@+=feaafiart1ev1aaatCvAUfKttLearuWrP9MDH5MBPbIqV92AaeXatLxBI9gBaebbnrfifHhDYfgasaacH8akY=wiFfYdH8Gipec8Eeeu0xXdbba9frFj0=OqFfea0dXdd9vqai=hGuQ8kuc9pgc9s8qqaq=dirpe0xb9q8qiLsFr0=vr0=vr0dc8meaabaqaciaacaGaaeqabaqabeGadaaakeaadaWcaaqaaiabdsgaKjabdEha3naaBaaaleaacqWGPbqAcqWGYbGCaeqaaOWaaeWaaeaacqWG0baDaiaawIcacaGLPaaaaeaacqWGKbazcqWG0baDaaGaeyypa0Jaemyta00aaSbaaSqaaiabdkhaYbqabaGcdaWadaqaaiabdgeabnaaBaaaleaacqWGPbqAaeqaaOGaeyOeI0Iaem4DaC3aaSbaaSqaaiabdMgaPjabdghaXbqabaGcdaqadaqaaiabdsha0bGaayjkaiaawMcaaiabgkHiTiabdEha3naaBaaaleaacqWGPbqAcqWGYbGCaeqaaOWaaeWaaeaacqWG0baDaiaawIcacaGLPaaaaiaawUfacaGLDbaadaahaaWcbeqaaiabikdaYaaaaaa@526D@
*w*_*iq*_(0) = *w*_*ir*_(0) = 0

The solution to the 2^nd ^equation, *w*_*ir*_(*t*), predicts the amount of double stranded DNA formed by re-annealing of previously synthesized single DNA strands. If *A*_*i *_denotes the amount of single strands of template, then (*A*_*i *_- *w*_*iq*_(*t*) - *w*_*ir*_(*t*)) reflects the amount of single template DNA available at time t within the i^th ^anneal step. The re-annealing process combines strands of opposite orientation, so that (*A*_*i *_- *w*_*iq*_(*t*) - *w*_*ir*_(*t*)) should be multiplied by a factor reflecting the amount of available anti-sense DNA available at time t. However, since we assumed the double-stranded DNA is formed from anti-sense strands at the same rate as from the template, there should always be the same amount of single-stranded DNA of both orientations present. For that reason, we square the factor denoting the amount of available single-stranded DNA of the template orientation. Each denaturation step is assumed to separate all duplexes, so that the amounts of *w*_*iq*_(0) and *w*_*ir*_(0) are zero at the start of each anneal step.

Since we assume all reactions go to equilibrium in the anneal/extensions step, we want the equilibrium solutions to the equations in Table 6 for each thermal cycle. Unfortunately, setting the derivatives equal to zero does not give a fully defined solution. The relative amount of double-stranded DNA formed by duplication as opposed to re-annealing is determined by the relative speed of the 2 modeled processes and can only be calculated by numerically integrating the differential equations until the solutions, *w*_*iq*_(*t*) and *w*_*ir*_(*t*), are very close to their respective equilibrium values, *w*_*iqE *_and *w*_*irE*_. Since equilibrium as characterized by our model occurs when all single-stranded DNA is incorporated into double-stranded DNA (either by duplication or by re-annealing), we numerically integrate the differential equation system until the term (*A*_*i *_- *w*_*iq*_(*t*) - *w*_*ir*_(*t*))^2 ^is less than 10^-12^. The solutions of the differential equations at that time are taken to be the equilibrium values for that time step.

The two rate constants, *M*_*q *_and *M*_*r *_do not change over the course of the amplification, but are even so not both identifiable, since, as long as both are large enough to ensure equilibrium is reached before the end of the anneal/extension step, only their relative sizes determine *w*_*iqE *_and *w*_*irE*_. Hence *M*_*q *_can be set equal to 1, leaving *M*_*r *_to be estimated by fitting the model to the fluorescent output.

From the assumptions encoded in Table 6, the amount of duplicated template at the end of the i^th ^anneal/extension step, *w*_*irE*_, is equal to the amount of probe and primer digested during the i^th ^anneal/extension step. Thus the predicted amounts of primer, probe and single-stranded amplicon available at the start of the i + 1^st ^anneal/extension step can be calculated as

Pi+1=Pi−wiqEQi+1=Qi−wiqEAi+1=Ai+1'=Ai+wiqE
 MathType@MTEF@5@5@+=feaafiart1ev1aaatCvAUfKttLearuWrP9MDH5MBPbIqV92AaeXatLxBI9gBaebbnrfifHhDYfgasaacH8akY=wiFfYdH8Gipec8Eeeu0xXdbba9frFj0=OqFfea0dXdd9vqai=hGuQ8kuc9pgc9s8qqaq=dirpe0xb9q8qiLsFr0=vr0=vr0dc8meaabaqaciaacaGaaeqabaqabeGadaaakqaabeqaaiabdcfaqnaaBaaaleaacqWGPbqAcqGHRaWkcqaIXaqmaeqaaOGaeyypa0Jaemiuaa1aaSbaaSqaaiabdMgaPbqabaGccqGHsislcqWG3bWDdaWgaaWcbaGaemyAaKMaemyCaeNaemyraueabeaaaOqaaiabdgfarnaaBaaaleaacqWGPbqAcqGHRaWkcqaIXaqmaeqaaOGaeyypa0Jaemyuae1aaSbaaSqaaiabdMgaPbqabaGccqGHsislcqWG3bWDdaWgaaWcbaGaemyAaKMaemyCaeNaemyraueabeaaaOqaaiabdgeabnaaBaaaleaacqWGPbqAcqGHRaWkcqaIXaqmaeqaaOGaeyypa0Jaemyqae0aa0baaSqaaiabdMgaPjabgUcaRiabigdaXaqaaiabcEcaNaaakiabg2da9iabdgeabnaaBaaaleaacqWGPbqAaeqaaOGaey4kaSIaem4DaC3aaSbaaSqaaiabdMgaPjabdghaXjabdweafbqabaaaaaa@5EEF@

The first cycle is started with the known initial amounts of primer and probe, *P*_0 _and *Q*_0_, as well as the unknown amount of initial amplicon, *A*_0_. Although *A*_0 _is not shown explicitly in the equations in Table 6, it is incorporated into all values of *w*_*iqE *_through the above equations. *A*_0 _is estimated by optimizing the fit of the model to the fluorescent data.

If *w*_*iqE *_estimates the amount of probe digested in the i^th ^cycle, the amount of probe digested throughout a PCR amplification of n cycles is estimated as Qused=∑i=1nwiqE
 MathType@MTEF@5@5@+=feaafiart1ev1aaatCvAUfKttLearuWrP9MDH5MBPbIqV92AaeXatLxBI9gBaebbnrfifHhDYfgasaacH8akY=wiFfYdH8Gipec8Eeeu0xXdbba9frFj0=OqFfea0dXdd9vqai=hGuQ8kuc9pgc9s8qqaq=dirpe0xb9q8qiLsFr0=vr0=vr0dc8meaabaqaciaacaGaaeqabaqabeGadaaakeaacqWGrbqudaWgaaWcbaGaemyDauNaem4CamNaemyzauMaemizaqgabeaakiabg2da9maaqahabaGaem4DaC3aaSbaaSqaaiabdMgaPjabdghaXjabdweafbqabaaabaGaemyAaKMaeyypa0JaeGymaedabaGaemOBa4ganiabggHiLdaaaa@4102@. Given this value, the calibration coefficient between total number of probe molecules digested and the total observed fluorescence over the entire amplification is calculated as

*Kf *= total observed fluorescence */Q*_*used*_

Note that since the amounts of probe and primer are given in nmol/L (in a 50 μl tubule), the estimate *Qused *is also in nmol/L. (See Appendix B(additional file 1) for the conversion formula between the concentration nmol/L in the 50 ml tubules and number of molecules.). The cost function minimized to fit the data is given below

∑i=thrn−1(Fi+1−Fi)∗(Fi−Kf∑k=1iwkqE)2,
 MathType@MTEF@5@5@+=feaafiart1ev1aaatCvAUfKttLearuWrP9MDH5MBPbIqV92AaeXatLxBI9gBaebbnrfifHhDYfgasaacH8akY=wiFfYdH8Gipec8Eeeu0xXdbba9frFj0=OqFfea0dXdd9vqai=hGuQ8kuc9pgc9s8qqaq=dirpe0xb9q8qiLsFr0=vr0=vr0dc8meaabaqaciaacaGaaeqabaqabeGadaaakeaadaaeWbqaamaabmaabaGaemOray0aaSbaaSqaaiabdMgaPjabgUcaRiabigdaXaqabaGccqGHsislcqWGgbGrdaWgaaWcbaGaemyAaKgabeaaaOGaayjkaiaawMcaaiabgEHiQmaabmaabaGaemOray0aaSbaaSqaaiabdMgaPbqabaGccqGHsislcqWGlbWsdaWgaaWcbaGaemOzaygabeaakmaaqahabaGaem4DaC3aaSbaaSqaaiabdUgaRjabdghaXjabdweafbqabaaabaGaem4AaSMaeyypa0JaeGymaedabaGaemyAaKganiabggHiLdaakiaawIcacaGLPaaadaahaaWcbeqaaiabikdaYaaaaeaacqWGPbqAcqGH9aqpcqWG0baDcqWGObaAcqWGYbGCaeaacqWGUbGBcqGHsislcqaIXaqma0GaeyyeIuoakiabcYcaSaaa@5980@

where F_*i *_denotes the observed (cumulative) fluorescence corresponding to the ith cycle, and there are n thermal cycles in the amplification. The optimization is organized as follows:

1) Initial guesses are made for *A*_0 _(unknown initial amount of amplicon) and *M*_*r *_(the rate constant for the re-annealing process).

2) Using those guesses, the model is run, ie, the differential equations are integrated for each anneal/extension step and the amounts of product, probe and primer updated between cycles.

3) At the end of the last cycle, the amount of probe used up by the amplification is calculated and equation 2 is used to compute the estimate for *Kf*.

4) Using the predicted *Kf *and the predicted equilibrium values for the solutions to the 1^st ^differential equation for each cycle, the cost function is evaluated.

5) An optimization routine is used to determine the next guess for *A*_0 _and *M*_*r*_.

Steps 2 – 5 are then repeated until the cost function is minimized.

The cost function describes weighted least squares estimation, since the squared difference between the observed fluorescence and the predicted fluorescence is multiplied by the i^th ^increment in fluorescence. We found that this weighting factor improved the fit by weighting more heavily the linear part of the sigmoid curve of cumulative fluorescence, which is the least affected by noise. To avoid contamination by the noise in the early cycles, we start the sum in the cost function with the first cycle (shown as *thr *in the above cost function) whose observed value is larger than 10 times the standard deviation of the observations over the first 10 cycles. Other thresholds could have been used, but this one was easily programmable.

## Authors' contributions

CJP conceived of the study and helped draft the manuscript. MVS developed the model. CRM provided valuable discussion and data. NJW provided early discussion pointing to the Taqman probe. MK provided insight and valuable discussions about the chemistry of the processes. All authors contributed in preparation of the final manuscript.

## Supplementary Material

Additional file 1 The Appendix to the paper as a Word document containing details of the expanded model described in the text, as well as the details of the estimation and optimization methods. Click here for file

## References

[B1] Gibson UE, Heid CA, Williams PM (1996). A novel method for real time quantitative RT-PCR. Genome Res.

[B2] Heid CA, Stevens J, Livak KJ, Williams PM (1996). Real time quantitative PCR. Genome Res.

[B3] Livak KJ, Schmittgen TD (2001). Analysis of relative gene expression data using real-time quantitative PCR and the 2(-Delta Delta C(T)) Method. Methods.

[B4] Rutledge RG, Cote C (2003). Mathematics of quantitative kinetic PCR and the application of standard curves. Nucleic Acids Res.

[B5] Higuchi R, Fockler C, Dollinger G, Watson R (1993). Kinetic PCR analysis: real-time monitoring of DNA amplification reactions. Biotechnology (N Y ).

[B6] Swillens S, Goffard JC, Marechal Y, de Kerchove EA, El Housni H (2004). Instant evaluation of the absolute initial number of cDNA copies from a single real-time PCR curve. Nucleic Acids Res.

[B7] Liu W, Saint D (2002). Validation of a quantitative method for real time PCR kinetics. Biochemical and Biophysical Research Communications.

[B8] Rutledge RG (2004). Sigmoidal curve-fitting redefines quantitative real-time PCR with the prospective of developing automated high-throughput applications. Nucleic Acids Res.

[B9] Goll R, Olsen T, Cui G, Florholmen J (2006). Evaluation of absolute quantitation by nonlinear regression in probe-based real-time PCR. BMC Bioinformatics.

[B10] Gevertz JL, Dunn SM, Roth CM (2005). Mathematical model of real-time PCR kinetics. Biotechnology and Bioengineering.

[B11] Mehra S, Hu WS (2005). A kinetic model of quantitative real-time polymerase chain reaction. Biotechnology and Bioengineering.

[B12] Wittwer CT, Herrmann MG, Moss AA, Rasmussen RP (1997). Continuous fluorescence monitoring of rapid cycle DNA amplification. Biotechniques.

