# Supplementary material for: Absolute estimation of initial concentrations of amplicon in a real-time RT-PCR process
Source: BMC Bioinformatics. 2007 Oct 23;8:409. doi: 10.1186/1471-2105-8-409 (PMC2194744; doi:10.1186/1471-2105-8-409)
Supplement: Additional file 1 — The Appendix to the paper as a Word document containing details of the expanded model described in the text, as well as the details of the estimation and optimization methods. [file 1471-2105-8-409-S1.doc]

Appendices

Appendix A. Extended models

*Duplication of template without fluorescent emission*

The anneal/extension step contains reactions in addition to the duplication of target sequence with fluorescent emission and re-annealing of existing target strands that comprise the basic model. For the data sets looked at for this paper with more primer available than probe molecules, including the duplication of target sequence without probe attachment and subsequent fluorescent emission led to an estimable model with improved estimates of the initial amount of amplicon. Including the extra parameter increases the need for enough observations to have the observed fluorescence reach the horizontal asymptote, but for the data and initial concentrations presented in this paper, 60 cycles proved sufficient.

The extended model is similar to the basic model discussed in the text and shown in Tables 1 and 2 with respect to notation and assumptions. The additional reaction is that of the primer attaching to the template without attachment of probe,

[A] + [P] ↔ [AP]

As before, the reaction attaching the primer to the template may be reversible, but is assumed to be followed quickly by the attachment of the Taq enzyme, after which extension is assumed to start irreversibly. A differential equation is added to the model for the added reaction, with the solution *w_is_(t)*, reflecting the amount of duplex formed from the template-primer complex, *AP*, ie without probe digestion. The extended model then looks like

Equation S1:

$$\begin{matrix} \frac{dw_{iq}\left(t\right)}{dt}=M_{q}\left[A_{i}-w_{iq}\left(t\right)-w_{is}(t)-w_{ir}\left(t\right)\right] \left[P_{i}-w_{iq}\left(t\right)-w_{is}\left(t\right)\right] \left[Q_{i}-w_{iq}\left(t\right)\right] \\ \frac{dw_{is}\left(t\right)}{dt}=M_{s}\left[A_{i}-w_{iq}\left(t\right)-w_{is}(t)-w_{ir}\left(t\right)\right] \left[P_{i}-w_{iq}\left(t\right)-w_{is}\left(t\right)\right] \\ \frac{dw_{ir}\left(t\right)}{dt}=M_{r}\left[A_{i}-w_{iq}\left(t\right)-w_{is}\left(t\right)-w_{ir}\left(t\right)\right]^{2} \\ w_{iq}\left(0\right)=w_{is}\left(0\right)=w_{ir}\left(0\right)=0 \end{matrix}$$

The added process is shown as the second equation*.* Note that its form is the same as the first equation, but without the *Q_i_ – w_iq_(t)* factor that indicated probe attachment. Other factors indicating single DNA strands, *Ai – w_iq_(t) – w_is_(t) – w_ir_(t),* and primers, *P_i_ – w_iq_(t) – w_is_(t),* are adjusted to reflect the added process using up single DNA strands and primer molecules. As was the case for the basic model, the equilibrium solutions of the PCR process are determined by the relative sizes of *M_r_, Mq* and M_s_, not by their absolute sizes. As long as all reactions complete in the cycle period (i.e. *Ai – w_iq_(t) – w_is_(t) – w_ir_(t)* is effectively zero) *M_q_* may be set equal to 1.0 without loss of generality. The differential equations in each cycle are evaluated until the solutions have converged to their equilibrium values, *wiqE,* *wisE*, and *wirE*. In practice the differential equation system is integrated until the expression (*Ai – w_iq_(t) – w_is_(t) – w_ir_(t)*)^2^ is below 10^-12^. This is the machine equivalent of there being no more single strands of DNA left. The amounts of probe, primer and available single DNA strands are updated similarly to the text from one thermal cycle to the next.

Equation S2:

*P_i+1_ = P_i_ - w_iqE_ - w_isE_*

*Q_i+1_ = Q_i_ – w_iqE_*

*A_i+1_ = A_i_ + w_iqE_+ w_isE_*

*Primer dimerization*

Primer dimerization was not a useful reaction to add for the data analyzed in this paper, but may be useful with different combinations of probe, primer and template. The equations used with the averaged Actin data were

Equation S3:

$$\begin{matrix} \frac{dw_{iq}\left(t\right)}{dt}=M_{q}\left[A_{i}-w_{iq}\left(t\right)-w_{ir}\left(t\right)\right] \left[P_{i}-w_{iq}\left(t\right)-w_{id}\left(t\right)\right] \left[Q_{i}-w_{iq}\left(t\right)\right] \\ \frac{dw_{id}\left(t\right)}{dt}=M_{d} \left[P_{i}-w_{iq}\left(t\right)-w_{id}\left(t\right)\right]^{2} \\ \frac{dw_{ir}\left(t\right)}{dt}=M_{r}\left[A_{i}-w_{iq}\left(t\right)-w_{ir}\left(t\right)\right]^{2} \\ w_{iq}\left(0\right)=w_{id}\left(0\right)=w_{ir}\left(0\right)=0 \end{matrix}$$

where the 2^nd^ equation corresponds to the primer dimerization. Note that we assume amounts of primers corresponding to DNA sequences of both orientations are equal. The equations below compute the amounts of single-stranded DNA, probe and primer available at the start of the i+1^st^ cycle using the model outputs from the previous cycle. The equations below correspond to the case that the primer-dimers extend during the extension step, so that the primers can not be used again.

Equation S4:

*P_i+1_ = P_i_ - w_iqE_ – w_idE_*

*Q_i+1_ = Q_i_ – w_iqE_*

*A_i+1_ = A_i_ + w_iq_*

Appendix B. Optimization

*Determining the starting cycle*

Either the basic or the expanded model is used to reflect the processes taking place in each thermal cycle and must be altered to reflect differences in experimental design. In this discussion we will use the phrase ‘target sequence’ to refer to the orientation of specific DNA sequence to which the probe molecules attach. If the initial solution in the reaction tubule contains only target sequence and no molecules of reverse orientation (as would be the case for transcription data, e.g.), then the first cycle will only produce the equivalent number of reverse orientation DNA sequence. In that case, the algorithm does not begin applying the model for prediction of either duplication or fluorescence with the first cycle but the second. If the initial solution contains equal numbers of both the target sequence and its reverse sequence, then the model is applied from the first cycle on.

*Conversion between nmol/L and molecule copy number*

The model is evaluated and optimized using the Matlab software from Mathworks. The starting concentrations of probe and primer molecules are assumed known (usually given in nmol/L) and are used by the program together with initial guesses for the parameter values M_r_ and A_0_ (and M_s_ if the expanded model is used). As a result of the units used for the probe and primer concentrations, the predicted increase in fluorescence and the estimated amount of initial amplicon are also found in units of nmol/L. However, since the amount of solution in a tubule is known (for the data presented in this paper, 50 μl of solution was always used), the concentrations in nmol/L are readily converted to molecular count by the following formula:

Equation S3:

$$\frac{Cnmol}{l}x\frac{Mole}{10^{9}nmol}x\frac{6.022x10^{23}molecules}{Mole}x\frac{l}{10^{6}\mu l}x\frac{50\mu l}{tubule}=C*3.011x10^{10}molecules$$

For each relevant thermal cycle, the set of differential equations must be integrated to the stopping point where the value of $(A_{i}-w_{iq}(t)-w_{is}(t)-w_{ir}(t))^{4}$ is indistinguishable from zero by the machine. At that point the steady state solution is assumed to have been reached, and the solution of the first differential equation (reflecting the key reaction of duplication of primer with digestion of probe and subsequent fluorescence) evaluated at that time is used as the predicted increase in fluorescence for the i^th^ cycle.
